# First Report of *Haplosporidium edule* Infection in the Olive-Green Cockle (*Cerastoderma glaucum*) from the Northern Adriatic Sea: Expanding Host Range and Geographic Distribution

**DOI:** 10.3390/pathogens15040415

**Published:** 2026-04-10

**Authors:** Alessia Vetri, Andrea Basso, Caterina D’Onofrio, Tobia Pretto, Edoardo Turolla, Federica Marcer, Eleonora Fiocchi, Giuseppe Arcangeli, Luana Cortinovis, Ewa Bilska-Zając, Vasco Menconi

**Affiliations:** 1 NRL Fish, Mollusc and Crustacean Diseases, Istituto Zooprofilattico Sperimentale delle Venezie, 35020 Legnaro, PD, Italy; avetri@izsvenezie.it (A.V.); cdonofrio@izsvenezie.it (C.D.); tpretto@izsvenezie.it (T.P.); efiocchi@izsvenezie.it (E.F.); garcangeli@izsvenezie.it (G.A.); lcortinovis@izsvenezie.it (L.C.); vmenconi@izsvenezie.it (V.M.); 2 Department of Animal Medicine, Production and Health MAPS, University of Padova, 35020 Legnaro, PD, Italy; federica.marcer@unipd.it; 3 Istituto Delta Ecologia Applicata srl, Centro Ricerche Molluschi, 44020 Goro, FE, Italy; veliger@istitutodelta.it; 4 Department of Parasitology and Invasive Diseases, Bee Diseases and Aquatic Animal Diseases, National Veterinary Research Institute, 24-100 Puławy, Poland; ewa.bilska@piwet.pulawy.pl

**Keywords:** haplosporidian parasites, mollusc diseases, molecular diagnostics, SSU rDNA, emerging diseases, histopathology, phylogenetic analysis

## Abstract

*Haplosporidium edule* is a haplosporidian parasite originally described in the common edible cockle (*Cerastoderma edule*) along the European Atlantic coast. In this study, we report the first detection of *H. edule* in the olive-green cockle (*Cerastoderma glaucum*) from the northern Adriatic Sea, representing both a novel host record and a new geographic occurrence. During a cross-sectional study conducted in May 2019, 90 *C. glaucum* specimens were collected from three lagoon sites in northeastern Italy. Histological examination of soft tissues revealed haplosporidian developmental stages, including plasmodia, sporoblasts and mature spores, within connective tissues of the mantle, digestive gland, gills and between gonadal tubules in eight individuals from the Goro Lagoon. Molecular characterization based on a fragment of the small subunit ribosomal DNA showed high similarity with the previously published *H. edule* sequence. Host identification was confirmed through *cytochrome c oxidase subunit I* barcoding together with morphological and histological analyses. These findings indicate that *H. edule* has a broader host range than previously recognized. Although prevalence was relatively low, the detection of this parasite in a new host species and geographic area highlights the importance of continued surveillance, particularly in the context of climate change, shellfish translocations and the expansion of aquaculture activities.

## 1. Introduction

Cockles are benthic bivalve molluscs belonging to the Cardiidae, which includes two morphologically similar and closely related species: the common edible cockle (*Cerastoderma edule*) and the olive-green cockle (*C. glaucum*), also reported in the previous literature as the “lagoon cockle” [1,2,3]. These species play a key ecological role, serving both as a vital food source and as connectors within trophic networks [4]. Additionally, they contribute to several ecosystem functions, including energy flow and carbon sequestration [5]. From a biological perspective, cockles have been thoroughly investigated [6] and are globally recognized as effective sentinel organisms and reliable bioindicators [7]. Furthermore, cockles represent an important natural marine resource that is traditionally harvested from the wild rather than farmed or cultured from spat [8]. Among them, the common edible cockle (*Cerastoderma edule*) stands out as one of the principal non-cultured bivalve species exploited in European coastal waters [9]. In the northern Adriatic Sea, cockles are mainly exploited at a local scale in lagoon environments (e.g., the Sacca di Goro), where they contribute to small-scale fisheries, although their economic importance remains limited compared to other bivalve resources [Edoardo Turolla, personal communication].

While their ranges partially overlap along the Atlantic European and Mediterranean coasts, *C. glaucum* predominantly inhabits brackish lagoons and estuaries, reflecting its broad tolerance to salinity fluctuations, whereas *C. edule* is mainly found in open coastal intertidal zones [10,11].

Beyond differences in habitat and distribution, both *Cerastoderma* species are susceptible to parasitic infections, particularly from haplosporidian protozoans [12,13]. Haplosporidians comprise a group of small protozoans that are widely distributed across aquatic environments [14]. Currently, more than 40 species have been described and assigned to four genera: *Haplosporidium*, *Minchinia*, *Bonamia* and *Urosporidium* [15]. Despite increasing research efforts, haplosporidians remain a poorly understood group and further studies of their diversity have led to the description of additional new species [16]. This limited knowledge underscores the importance of studying their life cycles, transmission pathways and host–parasite interactions, which are essential for advancing our understanding of haplosporidian ecology, epidemiology and their potential impacts on host populations.

Although the complete life cycle of haplosporidians has not yet been fully resolved, it generally includes multinucleate plasmodial stages and ovoid spores characterized by a wall and a polar orifice [17]. The morphology of spore ornamentation is a key taxonomic character used to distinguish among genera and species [14]. However, taxonomic boundaries can be blurred, as exemplified by *Bonamia perspora*, the only known *Bonamia* species that produces spores resembling those of the genus *Haplosporidium* [18,19]. Consequently, the morphological differentiation between these genera can be complicated and highlights the need for integrated histological and molecular approaches.

Epidemiological evidence suggests that haplosporidians may be transmitted directly between hosts, even in non-spore-forming species such as members of the genus *Bonamia* [20]. Moreover, the detection of haplosporidian DNA in water and sediment samples supports the hypothesis that free-living transmissible stages may exist or that intermediate hosts, including zooplankton, could be involved in their life cycles [21]. Haplosporidians infect a broad range of aquatic invertebrates and may also act as hyperparasites of other parasitic organisms, including trematodes, polychaetes and nematodes, many of which themselves parasitize bivalves [17,22].

Due to their ecological and economic significance, haplosporidian infections in molluscs and crustaceans are the most extensively studied. Notable examples include *Haplosporidium nelsoni* and *H. costale*, causative agents of MSX disease in the eastern oyster *Crassostrea virginica* [23,24,25,26], as well as *Bonamia ostreae* and *B. exitiosa*, which cause bonamiosis in flat oysters (*Ostrea* spp.) and are associated with mass mortality events, particularly in aquaculture settings [27,28,29,30,31,32]. Consequently, *B. ostreae* and *B. exitiosa* are subject to mandatory reporting within the European Union and to the World Organisation for Animal Health (WOAH).

In contrast, relatively few haplosporidian species have been reported in members of the family Cardiidae. *Minchinia tapetis* infections have been documented in *C. edule* from several locations, including the Netherlands [33], the United Kingdom [21,34], Ireland [35] and Spain [27]. *Minchinia mercenariae*-like parasites have been detected only in common edible cockles from the Ría de Arousa [22], while *Urosporidium* sp. has been observed as a hyperparasite of the turbellarian *Paravortex cardii* [36].

*Haplosporidium edule* was first described in *Cerastoderma edule* from Galicia, Spain, by Azevedo et al. (2003) [37]. However, the original description did not include molecular characterization (DNA sequencing and phylogenetic analyses) to support its taxonomic placement, nor did it provide a comprehensive histopathological assessment of host tissue alterations associated with infection. During routine histological examinations, multiple developmental stages of the parasite, including plasmodia, sporonts and sporoblasts, were observed in the digestive gland and adjacent connective tissues. Mature spores, measuring approximately 3 × 2 µm, are characterized by bifurcated projections and a folded spore wall. Since its initial description, *H. edule* has also been reported in *C. edule* populations from the Netherlands [33] and Wales [34]. In this context, the present study reports the first detection of *Haplosporidium edule* in a new host species, the olive-green cockle (*Cerastoderma glaucum*), and documents its occurrence in the Mediterranean Sea, thereby expanding both its known host range and geographic distribution.

## 2. Materials and Methods

### 2.1. Sample Collection

Specimens of cockle were collected in May 2019 from three sites in the northern Adriatic Sea during a cross-sectional study. Cockles were sampled from the Chioggia (45°13′ N 12°17′ E) and Porto Caleri Lagoons (45°09′ N 12°30′ E) using hydraulic dredges, while manual collection from the sediment surface was performed in the Goro Lagoon (44°50′ N 12°17′ E). These sites were selected due to their high productivity of molluscs, including *Ruditapes philippinarum*, *Mytilus galloprovincialis* and *Magallana gigas*, and because they are included in ongoing pathogen surveillance programs coordinated by competent authorities. During these monitoring activities, cockles are frequently found as bycatch together with clams. Given the co-occurrence of multiple bivalve species, these lagoons were considered particularly relevant for assessing the presence of pathogens, including those potentially transmissible between species.

Collected samples were stored at +4 °C in refrigerated containers and transported to the National Reference Laboratory for fish, mollusc and crustacean diseases at the Istituto Zooprofilattico Sperimentale delle Venezie (Padua, Italy) for subsequent analyses. In total, 30 viable individuals per site were collected for subsequent analyses. The shell length of the cockles was measured using a Digital Caliper (Mahr GmbH, Göttingen, Germany) and specimens ranging from 20 to 35 mm were subjected to histological and molecular analyses. Specimens were aseptically opened and for each specimen a transverse section of soft tissues including mantle, gills, gonads and digestive gland was fixed in Davidson’s solution for histological analysis. A second section of the same tissues was preserved in 96% ethanol and stored at +4 °C for molecular analyses.

### 2.2. Species Identification of Cockles

All specimens of *Cerastoderma glaucum* are morphologically distinguished from *C. edule* based on a combination of shell shape, ligament characteristics, dorsal profile and external sculpture, according to Turolla, 2007 [38].

An additional taxonomic feature for host species identification was provided by the histological examination of digestive tubule phases, following Carballal et al. 2016 [3]. *Cerastoderma glaucum* is characterized by a high proportion of digestive tubules in the breakdown phase, showing marked loss of epithelial integrity. Tubules in this phase exhibit extensive cellular degeneration, disappearance of most nuclei, brownish deposits and abundant cellular debris. This pattern is considered a species-specific physiological trait related to the digestive cycle rather than a pathological condition [3]. In contrast, the digestive gland of *C. edule* consists predominantly of synchronous digestive tubules in the digestive phase, with tall, well-organized digestive cells, nests of young basophilic cells and a reduced lumen. No epithelial disruption or digestive tubules in the breakdown phase were observed in *C. edule*. Following these morphological and histological traits, all collected specimens were taxonomically identified.

To ensure maximum taxonomic accuracy, molecular species confirmation was additionally performed on a subset of randomly selected specimens (five per site) to provide unequivocal taxonomic identification. In particular, abductor muscle tissue from these specimens was used for amplification of a fragment of the mitochondrial *cytochrome c oxidase subunit I (COI)* gene. Total DNA was extracted from tissues (see Section 2.4) and amplified using universal barcode primers targeting the *COI* gene [39]. The PCR reaction was performed in a total volume of 50 µL containing 1× buffer, 2.5 mM MgCl_2_, 0.5 µM of each primer, 1 mM dNTP mix, 2 U of Platinum™ Taq DNA Polymerase (Thermo Fisher Scientific, Waltham, MA, USA) and approximately 500 ng of template DNA. Amplification was performed with a GeneAmp^®^ PCR System 9700 thermal cycler (Applied Biosystems, Foster City, CA, USA) using the following thermal protocol: initial denaturation at 94 °C for 30 s; 35 cycles of denaturation at 94 °C for 30 s, annealing at 52 °C for 30 s and extension at 72 °C for 60 s; followed by a final extension at 72 °C for 10 min. PCR products were visualized and sequenced as described below. High-quality chromatograms were assembled into consensus sequences and queried against the Barcode of Life Data (BOLD) System using “animal: species” databases and “exhaustive search” mode and the GenBank database using the BLASTn algorithm for molecular species identification. Subsequently, specimens found positive for haplosporidian parasites through histological examination were subjected to additional confirmation of host taxonomy.

### 2.3. Histological Evaluation

Sections of *Cerastoderma glaucum* soft tissues, including the digestive gland, gonads, mantle and gills, were fixed in Davidson’s solution for 48 h. Following fixation, tissues were dehydrated through a graded ethanol series and embedded in paraffin. Serial sections of 3 μm thickness were cut and stained with Mayer’s Hematoxylin and Eosin-Y (H–E) for routine histopathological examination. Additional sections were alternatively stained with the Ziehl–Neelsen (Z–N) method using the Acid-Fast Bacteria (AFB) protocol on an ArtisanLink Pro automated histochemical stainer (Dako, Agilent Technologies, Santa Clara, CA, USA).

Microscopic examination was performed at 4×, 10×, 25×, 40× and 100× magnifications using a Nikon H550L microscope (Nikon Europe BV, Amsterdam, The Netherlands). Digital images were acquired with a Nikon DS-Ri2 camera (Nikon Europe BV) and processed using NIS-Elements BR software, Version 5.30.03 (Nikon Europe BV), to document the presence of parasites and any associated pathological alterations. Infection intensity was classified by histological examination using a semi-quantitative approach adapted from the qualitative criteria described by Carballal et al. [27]. Light infections were characterized by the presence of only a few parasites within tissues, whereas moderate infections involved larger tissue areas with a higher abundance of parasites. In the present study, an additional category of heavy infection was defined to describe cases with high parasite density, widespread distribution across all analyzed tissues, and the presence of multiple developmental stages (plasmodia, sporonts and sporoblasts).

Specimens showing putative haplosporidian infections in histology were then analyzed for molecular confirmation and phylogenetic relationship.

### 2.4. Molecular Detection and Phylogenetic Analysis of Haplosporidium edule

Samples from eight specimens found positive for putative haplosporidian parasites by histological evaluation were removed from ethanol and air-dried at room temperature on absorbent paper prior to DNA extraction. Genomic DNA was extracted separately from pooled tissues of each specimen (gills, mantle and digestive gland) to maximize the probability of detecting the parasite, given that its tissue distribution in *C. glaucum* is not fully characterized using the QIAamp^®^ DNA Mini Kit (QIAGEN, Hilden, Germany), following the manufacturer’s protocol. DNA quality and concentration were assessed using a NanoDrop Lite spectrophotometer (Thermo Fisher Scientific), yielding concentrations ranging from 180 to 250 ng/µL and A260/280 ratios between 1.8 and 1.9, with no significant differences observed among samples.

Extracted DNA from each sample was subjected to two different PCR amplifications. The first PCR was performed using the primers HAP-F1 and HAP-R3 [40], which amplify an approximately 350 bp fragment of the small subunit ribosomal DNA (SSU rDNA). The second PCR employed the haplosporidian-specific primer pair C5f-hap and sB1N, targeting all known haplosporidians [21] and yielding an amplicon of approximately 750 bp. Both primer pairs target regions of the SSU rDNA, a molecular marker widely used for haplosporidian detection and taxonomic identification [15,21,41,42,43]. The PCR reactions were performed in a total volume of 25 µL using the Platinum™ Taq DNA Polymerase kit (Thermo Fisher Scientific), largely following the reagent concentrations described in previous studies [21,40]. The only modification concerned the MgCl_2_ concentration, which was adjusted to 2.5 mM for the amplification using the C5f-hap and sB1N primers. Amplifications were carried out in a GeneAmp^®^ PCR System 9700 thermal cycler (Applied Biosystems) under the following conditions: an initial denaturation at 94 °C for 5 min; 35 cycles of denaturation at 94 °C for 30 s, annealing at 48 °C for 60 s (HAP-F1/HAP-R3) or 60 °C for 30 s (C5f-hap/sB1N) and extension at 72 °C for 60 s; followed by a final extension at 72 °C for 10 min.

PCR products were separated on a 1% agarose gel via electrophoresis at 110 V for 50 min and visualized with the Gel Doc™ EZ Imaging System (Bio-Rad Laboratories, Hercules, CA, USA). Samples amplified with the C5f-hap and sB1N primers that exhibited a single band at the expected size were selected for Sanger sequencing, because this amplicon was more suitable for species identification due to its greater length compared to the shorter PCR product. Sequencing was performed in both directions and the resulting chromatograms were manually inspected for quality. Consensus sequences were assembled after trimming the primer sequences and were subsequently queried against the GenBank database using the BLASTn algorithm.

To investigate phylogenetic relationships within the genus *Haplosporidium*, at least one SSU rDNA sequence from each accepted species was included in the analysis [17]. The multiple sequence alignment was performed using the MAFFT (v.7.526) online service [44]. To avoid alignment bias and redundancy, prior to phylogenetic analysis, longer sequences were trimmed to the length of the obtained consensus sequence, while sequences shorter than 600 nucleotides were excluded. Phylogenetic analysis was performed using a Maximum Likelihood approach implemented in IQ-TREE v.2.4.0 [45]. The best-fitting nucleotide substitution model was selected using ModelFinder v.2.2.0 implemented in the software [46]. Node support was assessed using the ultrafast bootstrap (UFB) method [47,48] with 10,000 replicates and only ultrafast bootstrap values ≥ 90 were reported. The tree was rooted using the haplosporidian sequence from *Haliotis iris* (AF492442) as the outgroup, as it represents a sister taxon to the genera *Urosporidium*, *Haplosporidium*, *Minchinia* and *Bonamia* [15,17].

## 3. Results

### 3.1. Species Identification of Cockles

**Figure 1:**
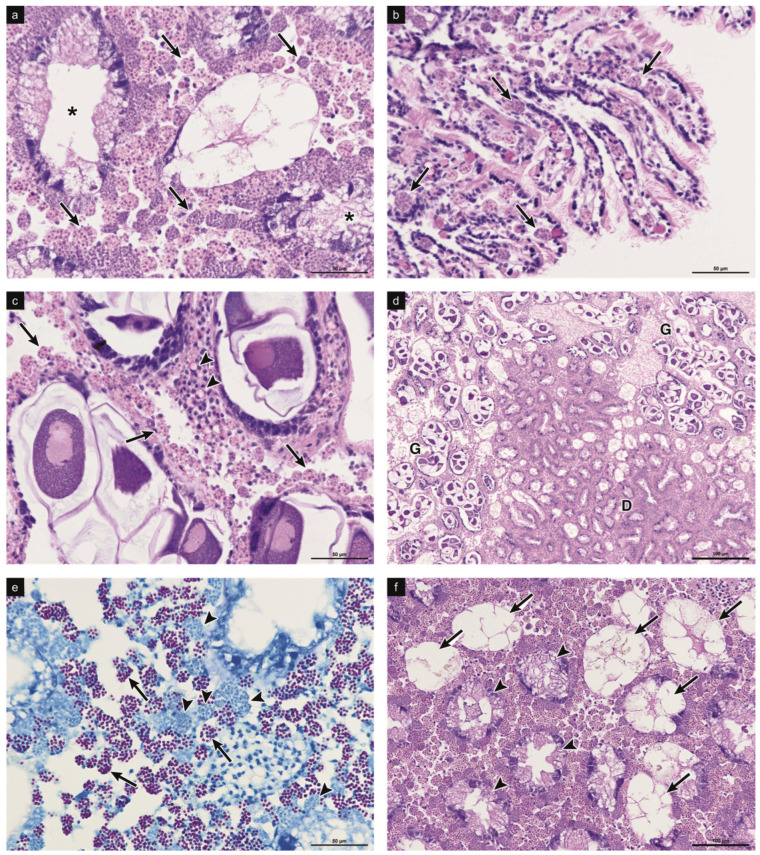
Light micrographs showing *Haplosporidium edule* infection in *Cerastoderma glaucum*. (**a**) Parasitic cells (arrow) are abundant in the connective tissues surrounding the digestive tubules (asterisk highlighting tubular lumen); H–E 40×. (**b**) Gills with evident *H. edule* parasitic cells (arrow); H–E 40×. (**c**) Gonadal tissue showing parasitic cells (arrow) and acidophilic hyalinocytes (arrowhead); H–E 40×. (**d**) A low-magnification image highlights the high intensity of haplosporidian infection within the visceral mass (D = digestive tubules; G = gonads); H–E 4×. (**e**) Ziehl–Neelsen staining allows the identification of mature spores (arrow), which stain red, while basophilic plasmodia stain blue (arrowhead); Z–N 40×. (**f**) Digestive tubules in breakdown phase (arrow) and in digestive phase (arrowhead) are shown; H–E 20×.

Morphological examination of all collected cockles confirmed the identification of *Cerastoderma glaucum*, consistent with previous taxonomic descriptions [11]. In lateral view, *C. glaucum* typically exhibited a subtriangular to subquadrate shell outline, in contrast to the broadly suboval and more equilateral shape characteristic of *C. edule*. The ligament in *C. glaucum* was consistently short and positioned closer to the umbo, whereas in *C. edule* it was relatively longer and extended further posteriorly. Dorsal profiles also differed notably: *C. glaucum* showed a more globose and inflated shell, while *C. edule* displayed a flatter, more elliptic dorsal outline. Shell sculpture further aided species differentiation. *Cerastoderma glaucum* possessed 20–22 radial ribs, typically smooth or only weakly ornamented, with wider interstices and a thicker, more robust periostracum. On the other hand, *C. edule* was characterized by 22–25 radial ribs bearing low, closely spaced scales, narrower interstices and a thinner periostracal layer. As reported by Carballal et al., 2016 [3], histological examination of the digestive gland revealed that in all individuals a high proportion of digestive tubules were observed in the breakdown phase, characterized by a marked loss of epithelial integrity. These tubules showed extensive cellular degeneration, disappearance of most nuclei and the presence of abundant cellular debris and brownish deposits within the tubule lumen. (Figure 1f).

These morphological distinctions were corroborated by molecular analyses. Amplification and sequencing of the *COI* gene confirmed that the 23 tested specimen (five per site and eight specimens positive for haplosporidians from the Goro Lagoon) were correctly identified as *Cerastoderma glaucum*, thereby validating the morphological classification and ruling out potential misidentification with the congeneric species *C. edule*. The obtained sequences were identical and showed ≥98% sequence coverage and nucleotide similarity with reference sequences, allowing unambiguous identification at the species level. The BOLD System identified the specimens as *C. glaucum* with a confidence of 99.25%, based on 42 supporting records, showing the highest similarity (99.83%) with BOLD ID GBMNC26301-20 (GenBank accession number MN613238), thus confirming the BLASTn results.

### 3.2. Histological Findings

A total of 90 specimens of *Cerastoderma glaucum* were examined across all sampling sites. Parasite stages consistent with the genus *Haplosporidium* were detected in eight specimens from the Goro Lagoon, corresponding to an infection prevalence of ~27% (8/30), while no infections were found at the other sites (0%).

Microscopic analysis revealed two distinct patterns of infection. Two specimens exhibited heavy infections, characterized by a high density of haplosporidian parasites (more than 40 parasites per microscopic field at 40× magnification), widely distributed across all examined connective tissues (digestive gland, gills, mantle and gonads). In these individuals, multiple developmental stages (plasmodia, sporonts and sporoblasts) were frequently observed intermingled in the connective tissues (Figure 1a–d).

In contrast, six specimens showed very light infections, with fewer than 10 parasites (mostly sporoblasts) per histological section, detected only sporadically and mainly restricted to the connective tissue surrounding the digestive gland.

In both patterns, parasite developmental stages up to mature spores appeared to be confined to the connective tissues. The host cellular response was minimal, with low hemocyte infiltration and no evidence of necrosis, edema or displacement of normal tissue. No mature spores were detected in the lumen of the digestive tubules.

The Ziehl–Neelsen staining proved effective in distinguishing between parasite stages: acid-fast mature spores stained red, while basophilic plasmodia retained the blue coloration of the counterstain (Figure 1e). This contrast allowed the clear identification and characterization of the sporulation stages.

No gross signs of infection or abnormal discoloration were observed in the soft tissues during gross examination.

### 3.3. Molecular Detection and Phylogenetic Analysis of Haplosporidium edule

All eight samples that tested positive for haplosporidian parasites by histological evaluation were also confirmed by molecular analysis, yielding amplicons of the expected size (approximately 350 bp) using the haplosporidian primers HAP-F1/HAP-R3 [40]. Using the C5f-hap and sB1N primers [21], amplification was obtained in all samples; however, only the heavily infected specimens yielded clear amplicons suitable for Sanger sequencing, whereas the other six samples produced faint bands and were therefore not suitable for sequencing. The quantity and quality of the total DNA extracted were comparable across all samples and are unlikely to have influenced PCR efficiency. Therefore, the observed differences between the two PCR assays are more likely due to lower parasite load or differences in primer efficiency and affinity, particularly for the longer amplicon. The two consensus sequences obtained from the amplified PCR products were identical, each with a length of 729 base pairs. A BLASTn search against the GenBank database (NCBI) revealed >99% nucleotide similarity with *Haplosporidium edule* (accession number DQ458793) infecting *Cerastoderma edule* and it was originally identified in specimens from the Burry Inlet, Wales. In both sequences, a transversion SNP was observed, with adenine (A) instead of thymine (T) present in the corresponding GenBank record. The SNP was consistently detected in both forward and reverse chromatograms and was not associated with background noise, indicating that it is unlikely to result from sequencing artefacts or polymerase errors.

**Figure 2:**
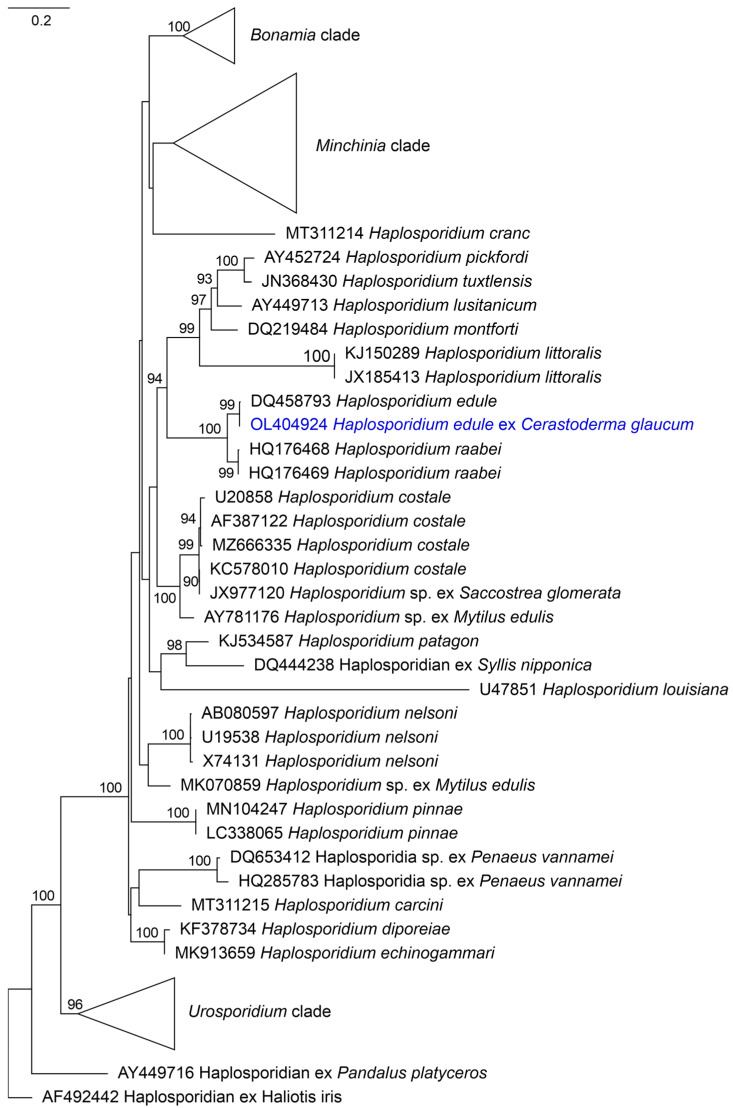
Maximum Likelihood phylogenetic tree (−log = 10,329.4749) based on SSU rDNA sequences. Values at the branches indicate ultrafast bootstrap support (>90). The tree was inferred using the TPM2+R4 nucleotide substitution model. Scale bar represents the number of nucleotide substitutions per site. The sequence in blue was obtained for the present study.

The refined dataset for phylogenetic analysis comprised 49 unique SSU rDNA sequences representing accepted *Haplosporidium* species (Table S1) and the alignment spanned 937 positions. Phylogenetic analysis was performed using the TPM2+R4 nucleotide substitution model, selected as the best-fitting model according to the Bayesian Information Criterion (BIC). The Maximum Likelihood tree (−log = 10,329.4749) clustered the obtained sequence within a well-supported clade (UFB = 99) containing other *H. edule* (DQ458793) retrieved from *C. edule* (Figure 2). Therefore, the phylogenetic analysis based on the SSU rDNA gene fragment further supported the taxonomic assignment, being consistent with the identification of the parasite as *H. edule*.

Due to the limited number of *H. edule* sequences currently available in public databases, the newly obtained sequences contribute valuable genetic data for future comparative and epidemiological studies. The sequence was submitted to GenBank under the accession number OL404924. These molecular findings, together with the histological evidence, confirm the presence of *H. edule* in *C. glaucum* from the northern Adriatic Sea, representing the first report of this parasite in this host species and region.

## 4. Discussion

This study expands the known host range of *Haplosporidium edule*, previously reported exclusively in the common edible cockle *Cerastoderma edule* [37], by documenting its occurrence in *C. glaucum* for the first time. The detection in this new host indicates that *H. edule* can infect multiple members of the genus *Cerastoderma*, reflecting the potential for host switching among closely related species when ecological barriers are reduced.

This finding also extends the known geographic distribution of the parasite, representing the first confirmed record in the Mediterranean Sea. Previously, *H. edule* had only been reported along the Atlantic coast, including Spain, the Netherlands and the United Kingdom, infecting *C. edule* [33,34,37]. The drivers of this new occurrence remain uncertain; however, environmental changes, including global climate shifts and the anthropogenic translocation of live molluscs via aquaculture or fisheries are recognized pathways for parasite dispersal [49], although direct evidence in this case is lacking.

In this framework, the distinct production intensity of the Goro Lagoon compared to the other investigated sites (Porto Caleri and Chioggia) should be considered when interpreting the spatial occurrence of *Haplosporidium edule*. The Goro Lagoon, located in the Po River Delta (northern Adriatic Sea), is widely recognized as one of the most productive bivalve farming areas in Italy and the Mediterranean, particularly for the Manila clam (*Ruditapes philippinarum*) [50,51], contributing substantially to national clam production [52]. High production levels are inherently associated with frequent movements of seed, juveniles and adult bivalves, both within the lagoon system and between geographically distant coastal areas. Such translocation practices are known to facilitate the introduction and spread of parasites and pathogens in marine bivalves, including haplosporidians, through the accidental transfer of infected hosts or free parasite stages [53,54]. Given that *C. edule* commonly co-occurs with commercially important bivalves and is widely harvested across Europe [9], accidental introduction via Manila clam seeding activities involving larvae or juveniles from other regions is plausible [55]. *Haplosporidium edule* may therefore have been introduced along with *C. edule* and subsequently transmitted to *C. glaucum* through cohabitation. In addition, environmental factors such as salinity, temperature and oxygen availability, which are known to influence haplosporidian infections [13], may also have contributed to the absence of the parasite in Chioggia and Porto Caleri. However, the lack of comparable environmental data among sites prevents a detailed assessment of their role in the present study.

The absence of mortality and signs of debilitation in the examined specimens, despite the wide tissue tropism of the parasite, suggests that *Haplosporidium edule*, under the current environmental conditions, is unlikely to pose an immediate threat to olive-green cockle populations in the investigated area. Furthermore, the lack of a marked hemocytic response supports the hypothesis that *H. edule* may presently behave as a commensal or opportunistic parasite in this host.

Histological analysis revealed a broader tissue distribution of *H. edule* in *C. glaucum* than previously reported in *C. edule*, with parasite stages present in the connective tissues of mantle, gill lamellae, digestive gland and between gonadal tubules, whereas in the original description they were confined to the digestive gland and adjacent connective tissues [37]. This pattern may reflect differences in host–parasite interactions or early infection dynamics. A similar distribution was observed by Ramilo et al., 2018 [22], for a *Minchinia mercenaria*-like parasite infecting *C. edule*, where uni- and binucleate stages and multinucleate plasmodia occurred in multiple tissues.

Molecular analysis identified a single transversion SNP in the SSU rDNA compared to the reference *H. edule* sequence. This variation may represent intraspecific genetic diversity, although the limited number of sequences currently available precludes determining whether it is associated with host shift, geographic isolation, or natural polymorphism. Additional sequences from diverse hosts and regions are needed to clarify the population structure of *H. edule*. The six specimens that produced faint PCR results corresponded to individuals displaying very light infections at histology, with only a few parasitic stages, mainly sporoblasts. This concordance between histological infection intensity and molecular outcome suggests that low parasite burden may have affected amplification efficiency.

The emergence of *H. edule* in a new host and ecosystem warrants attention, as haplosporidian parasites can cause disease outbreaks under favourable conditions or in distressed populations [56]. The expansion of *C. edule* into the Mediterranean also raises concerns about further introductions of parasites via live mollusc trade.

The present study represents a single cross-sectional investigation aimed at reporting the occurrence of *H. edule* in a new host species and geographic area. Although the histological analysis included 30 individuals per site, an adequate number to reliably detect and describe the parasite, this sample size does not allow for a robust assessment of prevalence or infection dynamics. Moreover, the reliance on a single sampling event prevents evaluation of seasonal or interannual variation. Importantly, this study provides the first histological description of *H. edule* in *C. glaucum*, representing a significant contribution to current knowledge. From a histopathological perspective, the number of examined specimens is sufficient to accurately characterize the parasite and its tissue distribution, even if limited for population-level inferences.

Future longitudinal studies with larger sample sizes will be essential to better characterize infection dynamics, prevalence patterns and potential pathogenic effects in *C. glaucum* populations. Overall, this work highlights the importance of continued monitoring of haplosporidian infections in Mediterranean bivalves to assess their ecological impact and support informed management strategies.

## 5. Conclusions

*Haplosporidium edule* is reported for the first time in *Cerastoderma glaucum* and the northeastern Mediterranean Sea. Despite its presence in multiple tissues (mantle, gill lamellae, digestive gland and gonadal tubules), infections were subclinical, with no macroscopic or histopathological lesions. The relatively low prevalence (~8.9%) and the lack of tissue response suggest that this represents a newly detected infection that currently behaves opportunistically in this novel host population. These findings underline the importance of ongoing surveillance to monitor the spread and ecological impact of *H. edule* and other pathogens investigating host susceptibility, environmental drivers and their potential introduction through live mollusc trade. The results provide a baseline for future comparative and epidemiological studies in Mediterranean bivalves.

## Data Availability

All data supporting the findings of this study are available within the article and its Supplementary Materials. The nucleotide sequence generated in this study has been deposited in the GenBank database under accession number OL404924.

## Supplementary Materials

The following supporting information can be downloaded at https://www.mdpi.com/article/10.3390/pathogens15040415/s1: Table S1. List of species and accession number used for phylogenetic analysis [15,19,22,24,37,41,42,57,58,59,60,61,62,63,64,65,66,67,68,69,70,71,72,73,74,75,76,77,78,79,80,81,82,83,84,85,86]. The sequence highlighted in bold was obtained for the present study. Specimens are reported in alphabetical order.
