# Supplementary material for: First Report of Haplosporidium edule Infection in the Olive-Green Cockle (Cerastoderma glaucum) from the Northern Adriatic Sea: Expanding Host Range and Geographic Distribution
Source: Pathogens. 2026 Apr 10;15(4):415. doi: 10.3390/pathogens15040415 (PMC13119199; doi:10.3390/pathogens15040415)
Supplement: Supplementary file 1 [file pathogens-15-00415-s001.zip › pathogens-4225404-supplementary.pdf]

Table S1: List of species and accession number used for phylogenetic analysis [15,19,22,24,37,41,42,57-86]. The sequence highlighted in bold was obtained for the present study. Specimens are reported in alphabetical order.

NCBI Accession	Species	Host(s)	Reference
GQ366703	*Bonamia exitiosa*	*Ostrea chilensis*	[57]
AF262995	*Bonamia ostreae*	*Ostrea edulis*	[58]
DQ356000	*Bonamia perspora*	*Ostrea equestris*	[19]
JF831803	*Bonamia* sp. ex *Dendostrea sandvicensis*	*Dendostrea sandvichensis*	[41]
AY449711	*Minchinia chitonis*	*Lepidochitona cinerea*	[59]
FJ518816	*Minchinia mercenariae*	*Mercenaria mercenaria*	[60]
MK070858	*Minchinia mytili*	*Mytilus edulis*	[15]
EF165631	*Minchinia occulta*	*Saccostrea cuccullata*	[61]
KY522823	*Minchinia* sp. ex *Cerastoderma edule*	*Cerastoderma edule*	[22]
AY449712	*Minchinia* sp. ex *Cyrenoida floridana*	*Cyrenoida floridana*	[62]
AY449710	*Minchinia tapetis*	*Ruditapes decussatus*	[63]
U20319	*Minchinia teredinis*	*Teredo* spp.	[64]
AY435093	Haplosporida sp. ex *Ruditapes decussatus*	*Ruditapes decussatus*	[65]
DQ653412	Haplosporidia sp. ex *Penaeus vannamei*	*Penaeus vannamei*	[66]
HQ285783	Haplosporidia sp. ex *Penaeus vannemei*	*Penaeus vannemei*	[67]
AF492442	Haplosporidian ex *Haliotis iris*	*Haliotis iris*	[68]
AY449716	Haplosporidian ex *Pandalus platyceros*	*Pandalus platyceros*	[69]
DQ444238	Haplosporidian ex *Syllis nipponica*	*Megasyllis nipponica*	[70]
MT311215	*Haplosporidium carcini*	*Carcinus maenas*	[42]
U20858<br>AF387122<br>KC578010<br>MZ666335	*Haplosporidium costale*	*Crassostrea virginica*	[71]
MT311214	*Haplosporidium cranc*	*Carcinus maenas*	[42]
KF378734	*Haplosporidium diporeiae*	*Diporeia* spp.	[72]
MK913659	*Haplosporidium echinogammari*	*Marinogammarus marinus, Pontogammarus robustoides*	[73]
DQ458793<br>**OL404924**	*Haplosporidium edule*	*Cerastoderma edule*<br>***Cerastoderma glaucum***	[37]
KJ150289<br>JX185413	*Haplosporidium littoralis*	*Carcinus maenas*	[74]
U47851	*Haplosporidium louisiana*	*Panopeus herbstii*	[75]
AY449713	*Haplosporidium lusitanicum*	*Patella pellucida*	[76]
DQ219484	*Haplosporidium montforti*	*Haliotis tuberculata*	[77]

X74131<br>AB080597<br>U19538	*Haplosporidium nelsoni*	*Crassostrea virginic, C. gigas*	[24]
KJ534587	*Haplosporidium patagon*	*Siphonaria lessonii*	[78]
AY452724	*Haplosporidium pickfordi*	*Physella gyrina, Lymnaea stagnalis, Planorbella campanulata*	[79]
MN104247<br>LC338065	*Haplosporidium pinnae*	*Pinna nobilis*	[80]
HQ176468<br>HQ176469	*Haplosporidium raabei*	*Dreissena polymorpha*	[81]
MK070859	*Haplosporidium* sp. ex *Mytilus edulis*	*Mytilus edulis*	[15]
AY781176	*Haplosporidium* sp. ex *Mytilus edulis*	*Mytilus edulis*	[82]
JX977120	*Haplosporidium* sp. ex *Saccostrea glomerata*	*Saccostrea glomerata*	[83]
JN368430	*Haplosporidium tuxtlensis*	*Siphonaria pectinata*	[84]
U47852	*Urosporidium crescens*	*Microphallus nicolli* parasite of *Callinectes sapidus*	[85]
AY449714	*Urosporidium* sp. ex *Stictodora lari*	*Stictodora lari* parasite of *Batillaria australis*	[62]
MN211513	*Urosporidium tapetis*	*Parvatrema duboisi* parasite of *Ruditapes philippinarum*	[86]
